# Supplementary material for: Floral presence and flower identity alter cereal aphid endosymbiont communities on adjacent crops
Source: J Appl Ecol. 2023 May 10;60(7):1409–23. doi: 10.1111/1365-2664.14426 (PMC11005096; doi:10.1111/1365-2664.14426)
Supplement: Supplementary file 1 — Table S1. Primers, and sequences used to identify endosymbionts of cereal aphids. Figure S1. Field experiment: effects of field variables on the aphid symbiont species. Figure S2. Outdoor pot experiment: the effect of flower treatment on aphid symbiont species. Figure S3. Outdoor pot experiment: the relationship between natural enemy diversity and symbiont diversity, over flower treatment and time. [file JPE-60-1409-s001.pdf]

SUPPORTING INFORMATION: Appendix 1

Floral presence and flower identity alter cereal aphid endosymbiont communities on adjacent crops

Sharon E. Zytynska[1,2*], Sarah Sturm[2], Cathy Hawes[3], Wolfgang W Weisser[2], Alison Karley[3]

[1] *Department of Evolution, Ecology, and Behaviour. Institute of Infection, Veterinary and Ecological Sciences. University of Liverpool, Crown Street, Liverpool, L69 7ZB, UK*

[2] *Technical University of Munich, Terrestrial Ecology Research Group, Department of Life Science Systems, School of Life Sciences, Hans-Carl-von-Carlowitz-Platz 2, 85354 Freising, Germany*

[3] *Ecological Sciences Department, The James Hutton Institute, Invergowrie, Dundee, DD2 5DA, UK*

* corresponding author email: *sharon.zytynska@liverpool.ac.uk*

Table S1: Primers, and sequences used to identify endosymbionts of cereal aphids

Figure S1. Field experiment: effects of field variables on the aphid symbiont species

Figure S2. Outdoor pot experiment: the effect of flower treatment on aphid symbiont species

Figure S3. Outdoor pot experiment: the relationship between natural enemy diversity and symbiont diversity, over flower treatment and time.

Table S1: Primers, and sequences used to identify endosymbionts of cereal aphids

Symbiont Species	Forward	sequence	Reverse	sequence
Buchnera symbiotica	Buch16S1F	GAGCTTGCTCTCTTTGTCGGCAA	Buch16S1R	CTTCTGCGGGTAACGTCACGAA
Hamiltonella defensa	10F	AGTTTGATCATGGCTCAGATTG	T419R	AAATGGTATTCGCATTTATCG
Regiella insecticola	10F	AGTTTGATCATGGCTCAGATTG	U443R	GGTAACGTCAATCGATAAGCA
Serratia symbiotica	10F	AGTTTGATCATGGCTCAGATTG	R443R	CTTCTGCGAGTAACGTCAATG
Fukatsuia symbiotica	10F	AGTTTGATCATGGCTCAGATTG	X420R	GCAACACTCTTTGCATTGCT
Rickettsia	16SA1	AGAGTTTGATCMTGGCTCAG	Rick16SR	CATCCATCAGCGATAAATCTTTC
Spiroplasma	10F	AGTTTGATCATGGCTCAGATTG	TKSSsp	TAGCCGTGGCTTTCTGGTAA
Rickettsiella	RCL16S-211F	GGGCCTTGCGCTCTAGGT	RCL16S-470R	TGGGTACCGTCACAGTAATCGA
Wolbachia	W-SpecF	CATACCTATTCGAAGGGATAG	W-SpecR	AGCTTCGAGTGAAACCAATTC
Arsenophonus	Ars-23S1F	CGTTTGATGAATTCATAGTCAAA	Ars-23S2R	GGTCCTCCAGTTAGTGTTACCCAAC
aphid COI	LCO1490	GGTCAACAAATCATAAAGATATTGG	HCO2198	TAAACTTCAGGGTGACCAAAAAAT

**Master Mix**	µl per reaction
DNA	2.0
5x Bioline MyTaq Reaction Buffer	4.0
PrimerF (20µM)	0.4
PrimerR (20µM)	0.4
Bioline MyTaq (5U/µl)	0.2
ddH_2O	13.0
total	20.0

**Touchdown PCR program 65-55°C**		
94°C	05:00	
94°C	00:15	10x temp **-1°C/**cycle
65-55°C	00:30	
72°C	00:30	
94°C	00:15	25x
55°C	00:30	
72°C	00:30	
72°C	06:00	
4°C	∞	

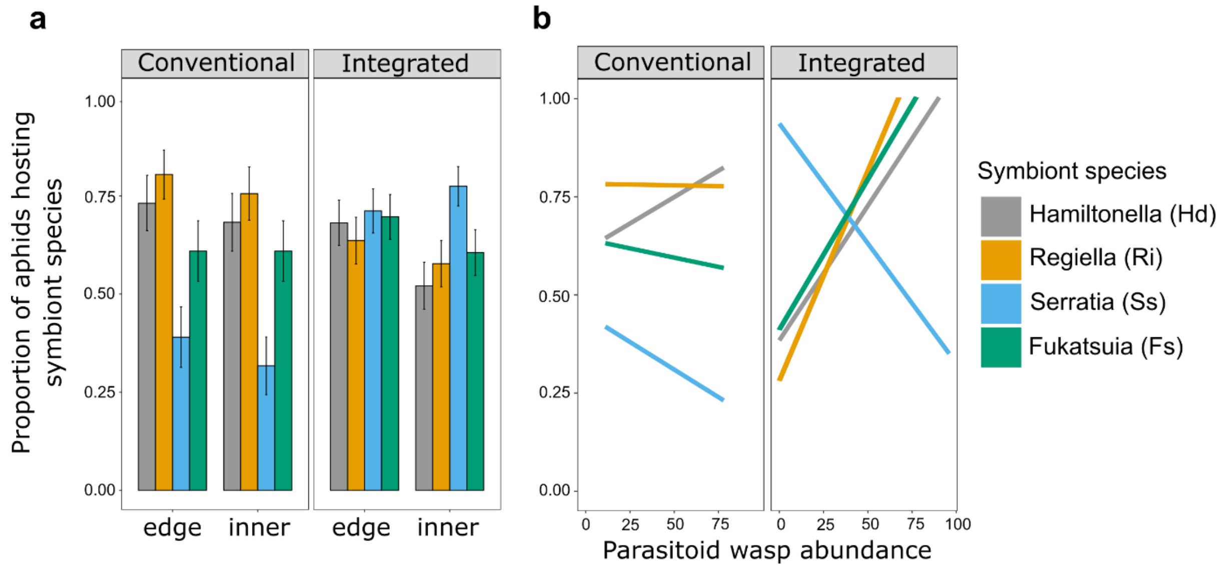

Fig S1. Field experiment: effects of field variables on the aphid symbiont species. The proportion of aphids hosting the different symbiont species within integrated and conventionally managed fields across (a) distance into the field (edge, 5-15m or inner, 30-50m), and (b) parasitoid wasp abundance. Aphid host multiple endosymbionts and therefore sum of proportions will be more than one. Error bars represent ±1SE.

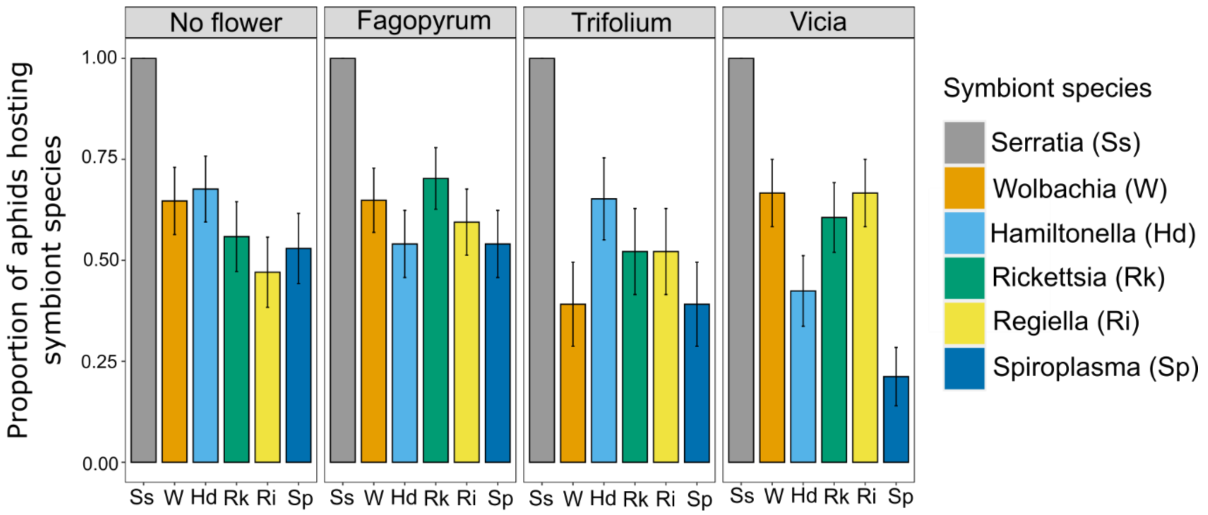

Fig S2. Outdoor pot experiment: the effect of flower treatment on aphid symbiont species. The proportion of aphids hosting different symbiont species across flower treatments. Error bars represent ±1SE.

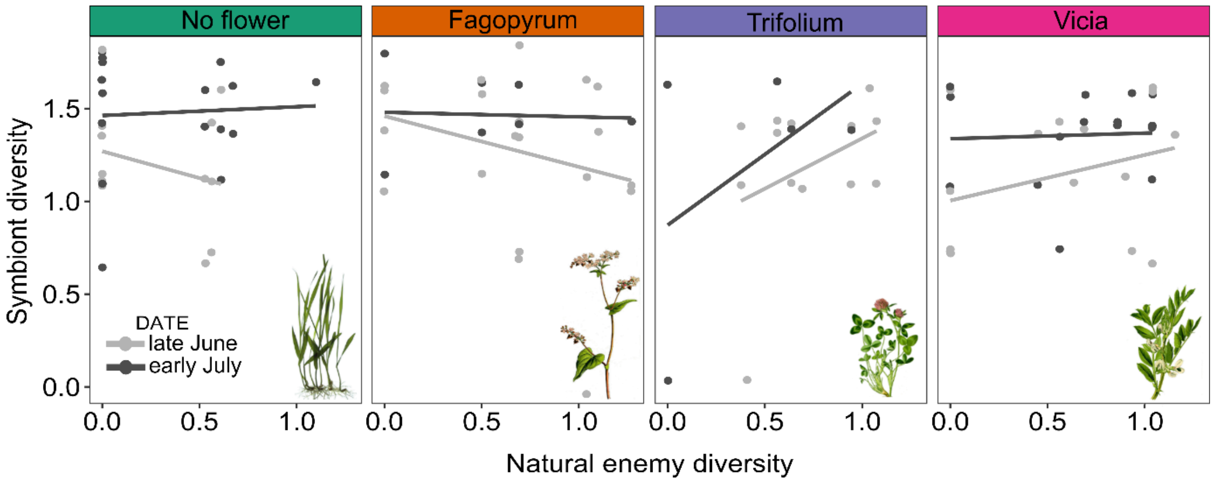

Fig S3. Outdoor pot experiment: the relationship between natural enemy diversity and symbiont diversity, over flower treatment and time. Only data for late June (n=71 aphids on 50 plants) and early July (n=53 aphids on 32 plants) shown in (c) due to lack of sufficient data for comparisons in late July (n=21 aphids on 12 plants).
